# Enhanced Degradation of Norfloxacin Under Visible Light by S-Scheme Fe_2_O_3_/g–C_3_N_4_ Heterojunctions

**DOI:** 10.3390/molecules29215212

**Published:** 2024-11-04

**Authors:** Guang Lu, Wei Li, Zheng Li, Guizhou Gu, Qiuju Han, Jiling Liang, Zhen Chen

**Affiliations:** 1 School of Civil Engineering, Liaoning Petrochemical University, Fushun 113001, China; luguang20121101@126.com (G.L.);; 2 School of Environmental & Safety Engineering, Liaoning Petrochemical University, Fushun 113001, China; 3 School of Petrochemical Engineering, Liaoning Petrochemical University, Fushun 113001, China; 4 School of Environmental and Chemical Engineering, Shenyang Ligong University, Shenyang 110159, China

**Keywords:** Fe_2_O_3_, g–C_3_N_4_, heterojunction, photocatalytic degradation, NOR

## Abstract

S-scheme Fe_2_O_3_/g–C_3_N_4_ heterojunctions were successfully fabricated by the ultrasonic assistance method to remove norfloxacin (NOR) under visible light irradiation. The synthesized catalysts were well studied through various techniques. The obtained Fe_2_O_3_/g–C_3_N_4_ heterojunctions exhibited an optimal photocatalytic degradation of 94.7% for NOR, which was 1.67 and 1.28 times higher than using Fe_2_O_3_ and g–C_3_N_4_ alone, respectively. In addition, the kinetic constant of NOR removal with Fe_2_O_3_/g–C_3_N_4_ composites was about 0.6631 h^−1^, and NOR photo-deegradation was still 86.7% after four cycles. The enhanced photocatalytic activity may be mainly attributed to the formation of S-scheme Fe_2_O_3_/g–C_3_N_4_ heterojunctions with built-in electric fields, which were beneficial to the separation and transfer of photostimulated charge carriers. Furthermore, a possible photo-degradation mechanism of NOR for S-scheme Fe_2_O_3_/g–C_3_N_4_ heterojunctions is described.

## 1. Introduction

Large amounts of antibiotics are used to treat bacterial infections in humans and animals [1]. Furthermore, antibiotics are widely applied as growth promoters in animal husbandry, agriculture and the pharmaceutical industry to prevent crop failure and economic losses from bacterial strains [2]. Unfortunately, only a small amount of antibiotics are metabolized by animals and humans, and most antibiotics are discharged into the environment, resulting in the global presence of antibiotics in the environment that affect the ecosystem and human health [3]. Take norfloxacin (NOR) for example, an antibiotic with recorded concentrations in the range of 0.251–0.470 mg∙L^−1^, 1.8–47.4 μg∙L^−1^ and 0.004–520 μg∙L^−1^ in surface water in India, Kenya and China, respectively [4,5,6,7]. Therefore, there is a tremendous need to develop a cost-effective technique for removing NOR from the environment. Among many treatment approaches, semiconductor-based photocatalysis is considered one of the most successful approaches for removing NOR-contaminated water owing to its ability to utilize solar energy [8,9].

Graphitic carbon nitride (g–C_3_N_4_), a non-metallic polymeric material, has been considered as a competent photocatalyst due to its low cost, suitable bandgap structure, excellent stability, simple preparation and non-toxic nature [10,11,12]. Nevertheless, its photocatalytic activity is limited owing to its narrow visible light response, low density of reactive sites, small specific surface areas and poor separation of photogenerated carriers [13,14,15]. One efficient approach to address these challenges is the construction of a Step-scheme (S-scheme) heterojunction between g–C_3_N_4_ and a well-matched semiconductor to generate an internal electric field, which could effectively promote photogenerated charge separation and boost photocatalytic performance [16,17,18]. For instance, C. Chen et al. prepared novel biochar-decorated Bi_4_O_5_Br_2_/g–C_3_N_4_ S-scheme heterojunctions for NOR degradation and found that the 5%–Bi_4_O_5_Br_2_/g–C_3_N_4_/C heterojunctions exhibited excellent photocatalytic degradation activity toward NOR, degrading 92.5% of the NOR within 72 min under visible light irradiation. However, the preparation of Bi_4_O_5_Br_2_/g–C_3_N_4_ S-scheme heterojunctions is rather high [19]. Hematite (Fe_2_O_3_) is a n–type reduction photocatalyst (RP) with excellent absorption in the visible light region and suitable E_CB_ and E_VB_ band positions, which can combine with g–C_3_N_4_ to form an S-scheme heterojunction structure to more quickly and effectively transfer photogenerated charges and promote their separation [20,21]. For instance, D.Q. Meng et al. prepared α–Fe_2_O_3_/g–C_3_N_4_ composites by the solvothermal method to degrade tetracycline molecules in wastewater and found that it exhibited a photocatalytic efficiency of 32.5% for tetracycline within 120 min [22]. Z. Li et al. prepared Fe_2_O_3_/g–C_3_N_4_ nano-heterostructures by combining co-precipitation and calcination methods, and their results showed optimal photocatalytic degradation of NOR (72.3%) [23]. From the above results, it is observed that the preparation method of the photocatalyst greatly impacts the antibiotic photo-degradation efficiency. Therefore, an ultrasonic assistance method was applied in this work to prepare Fe_2_O_3_/g–C_3_N_4_ composites to improve the removal efficiency of NOR.

In this paper, the magnetically separable Fe_2_O_3_/g–C_3_N_4_ composites were, firstly, designed by an ultrasonic assistance method to photo-degrade NOR molecules in wastewater under visible light irradiation. The results showed that Fe_2_O_3_/g–C_3_N_4_ heterojunctions exhibited superior photocatalytic degradation capabilities compared to Fe_2_O_3_ or g–C_3_N_4_ alone due to the built-in electric field in Fe_2_O_3_/g–C_3_N_4_ composites. Additionally, a possible S-scheme photocatalytic mechanism for NOR molecules by Fe_2_O_3_/g–C_3_N_4_ composites was described.

## 2. Results and Discussion

### 2.1. Crystal Structures

**Figure 1:**
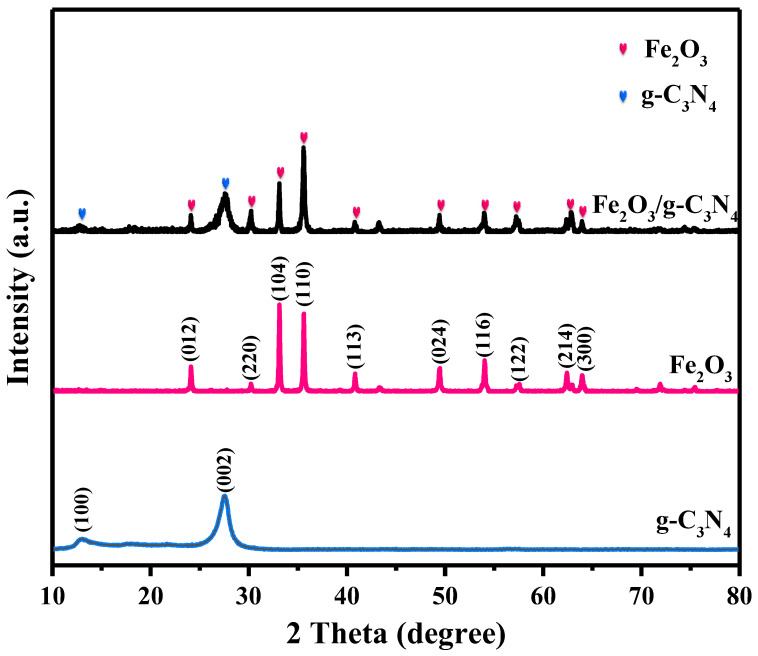
XRD patterns of the different catalysts.

The crystal structure of as-synthesized g–C_3_N_4_, Fe_2_O_3_ and Fe_2_O_3_/g–C_3_N_4_ samples were determined by XRD patterns. As shown in Figure 1, g–C_3_N_4_ alone has two characteristic peaks at 12.98° and 27.56°, which corresponds to the (100) and (002) planes, respectively (JCPDS NO. 87-1526) [24]. α–Fe_2_O_3_ alone exhibits characteristic peaks at 24.14°, 30.26°, 33.15°, 35.65°, 40.89°, 49.46°, 54.09°, 57.35°, 62.44° and 63.99°, which corresponds to the (012), (220), (104), (110), (113), (024), (116), (122), (214) and (300) planes, respectively (JCPDS NO. 33-0664) [25]. The positions of the characteristic peaks in the Fe_2_O_3_/g–C_3_N_4_ composite match well with Fe_2_O_3_ and g–C_3_N_4_ individually, indicating the successful synthesis of the heterojunctions in the Fe_2_O_3_/g–C_3_N_4_ composites. In addition, the ratio of intensities of reflections (104) and (110) for the Fe_2_O_3_ phase in the mixture significantly changed, suggesting that Fe_2_O_3_ in the composite samples developed with a preferred orientation of (104).

### 2.2. Morphology

**Figure 2:**
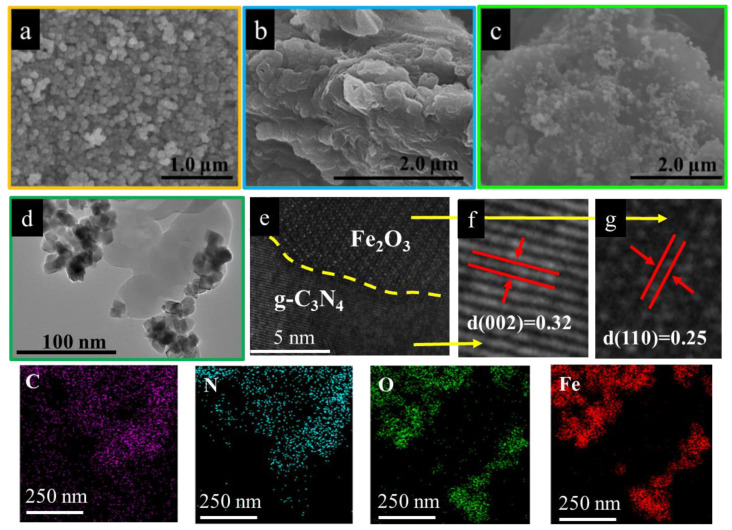
SEM diagram. of Fe_2_O_3_ (**a**), g–C_3_N_4_ (**b**) and Fe_2_O_3_/g–C_3_N_4_ (**c**). TEM (**d**), HRTEM (**e**–**g**) and corresponding element mapping of the Fe_2_O_3_/g–C_3_N_4_ composite.

The surface morphology of Fe_2_O_3_, g–C_3_N_4_ and Fe_2_O_3_/g–C_3_N_4_ was analyzed by SEM and TEM. As shown in Figure 2a–c, the g–C_3_N_4_ sample displays an irregular, layered structure, whereas the Fe_2_O_3_ sample is made up of numerous small uniform particles with a size of about 200 nm. After the coupling of Fe_2_O_3_ and g–C_3_N_4_, the Fe_2_O_3_/g–C_3_N_4_ heterojunction is formed by Fe_2_O_3_ particles fixed onto the layered structure of g–C_3_N_4_. The TEM image of Fe_2_O_3_/g–C_3_N_4_ (as shown in Figure 2d) indicates that many dark nano-aggregates of Fe_2_O_3_ particles are dispersed onto the surface of transparent g–C_3_N_4_ plates. As shown in Figure 2e, a distinct and close interface is formed between Fe_2_O_3_ and g–C_3_N_4_. The HRTEM images of Fe_2_O_3_/g–C_3_N_4_ (as shown in Figure 2f,g) exhibit lattice spacing of 0.25 and 0.32 nm, which correspond to the (110) crystal plane of Fe_2_O_3_ and the (002) crystal plane of g–C_3_N_4_, respectively. Element mapping of Fe_2_O_3_/g–C_3_N_4_ was also conducted, as shown in Figure 2, and revealed the presence of the elements C, N, Fe and O in the Fe_2_O_3_/g–C_3_N_4_ heterojunction photocatalyst. This confirmed the successful formation of the Fe_2_O_3_/g–C_3_N_4_ heterojunction photocatalyst.

### 2.3. XPS Analysis

**Figure 3:**
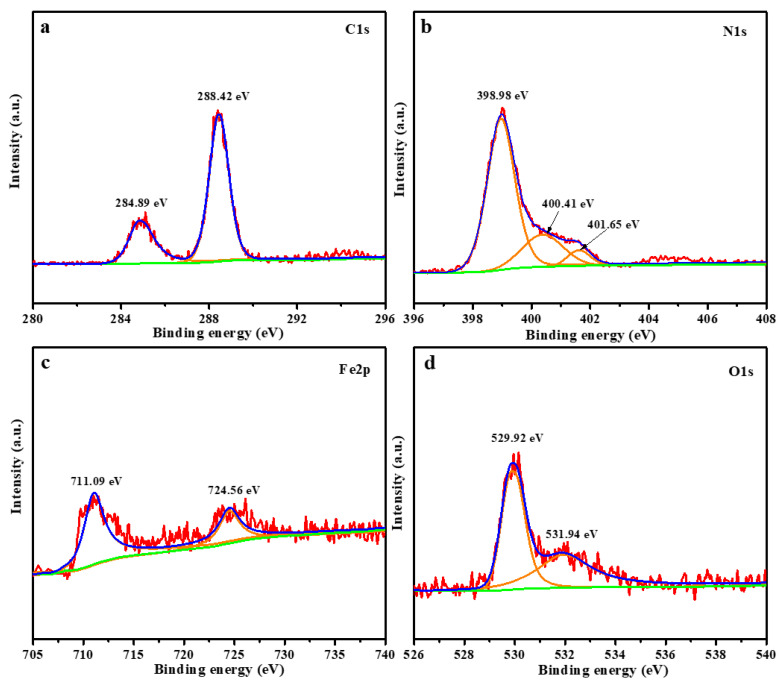
XPS spectra of the Fe_2_O_3_/g–C_3_N_4_ composite; (**a**) C 1s, (**b**) N 1s, (**c**) Fe 2p and (**d**) O 1s.

The compositions and chemical bonding states of the Fe_2_O_3_/g–C_3_N_4_ composites were investigated by X-ray photoelectron spectroscopy (XPS). As shown in Figure 3a, the C 1s spectrum of the Fe_2_O_3_/g–C_3_N_4_ sample can be fitted into two peaks at 284.89 and 288.42 eV, which are attributed to sp2-hybridized C in C–C and N–C=N, respectively [26,27]. As shown in Figure 3b, the N 1s spectrum of the Fe_2_O_3_/g–C_3_N_4_ sample can be divided into three peaks at binding energies of 398.98, 400.41 and 401.65 eV, which belong to sp2-bonded N in C=N–C, C–N_3_ and C–N–H, respectively [28,29]. The Fe 2p spectrum of the Fe_2_O_3_/g–C_3_N_4_ sample (as shown in Figure 3c) contains peaks at 711.09 and 724.56 eV, which are attributed to the Fe 2p_3/2_ and Fe 2p_1/2_ of Fe^3+^ in Fe_2_O_3_, respectively [30,31]. The O 1s spectrum of the Fe_2_O_3_/g–C_3_N_4_ sample (as shown in Figure 3d) shows two main peaks, at 529.92 and 531.94 eV, which belong to the lattice oxygen of Fe_2_O_3_ and the •OH group on the surface of the composite, respectively [32]. The XPS results further confirm the formation of the Fe_2_O_3_/g–C_3_N_4_ heterojunction.

### 2.4. Optical Properties

**Figure 4:**
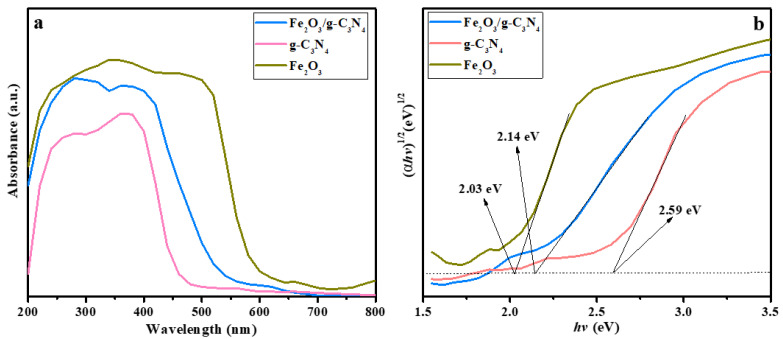
UV–vis DRS spectra (**a**) and Tauc diagram (**b**) of all samples.

The optical absorption capability of the different samples was tested by UV–vis diffuse reflectance spectroscopy (DRS). It can be seen from Figure 4a that both g–C_3_N_4_ and Fe_2_O_3_ can absorb UV and visible light, and their light absorption edges are approximately 480 and 696 nm, respectively. The Fe_2_O_3_/g–C_3_N_4_ composite shows a significantly red-shift compared to pure g–C_3_N_4_, and the absorption intensity in both UV and visible light is enhanced.

The band gap energy of the as-prepared catalysts can be obtained using the following equation:

*αhv* = *A*(*hv* − *E_g_*)^*n*/2^ (1)

where *Eg*, *α*, *h*, *v* and *A* represent the band gap energy, the absorption coefficient, Planck’s constant, the light frequency and a constant, respectively. As g–C_3_N_4_ and Fe_2_O_3_ are the indirect band gap semiconductors, the *n* value of g–C_3_N_4_ and Fe_2_O_3_ is four [33]. As shown in Figure 4b, two different slopes related to different transitions were discovered, and the sharp one is used to estimate the *Eg* value. Therefore, the *Eg* of g–C_3_N_4_, Fe_2_O_3_ and Fe_2_O_3_/g–C_3_N_4_ samples is about 2.59, 2.03 and 2.14 eV, which are drawn systematically by considering the baseline (the dotted line in Figure 4b).

In addition, the conduction and valence potentials of the g–C_3_N_4_ and Fe_2_O_3_ samples could be calculated based on the Mulliken electronegativity theory shown below:

*E_VB_* = *χ* − *E_e_* + 0.5*E_g_* (2)

*E_CB_* = *E_VB_* − *E_g_* (3)

where χ represents the absolute electronegativity of the semiconductor (χ value of g–C_3_N_4_ and Fe_2_O_3_ is 4.22 and 5.87 eV [34], respectively). *E_e_* is the free electron energy on the hydrogen scale (approximately 4.5 eV). According to Equation (2), the valence band potentials (*E_VB_*) of g–C_3_N_4_ and Fe_2_O_3_ were counted to be 1.01 and 2.39 eV, respectively. According to Equation (3), the conduction band potentials (*E_CB_*) of g–C_3_N_4_ and Fe_2_O_3_ were calculated to be −1.12 and 0.48 eV, respectively.

### 2.5. Charge Separation and Transfer

**Figure 5:**
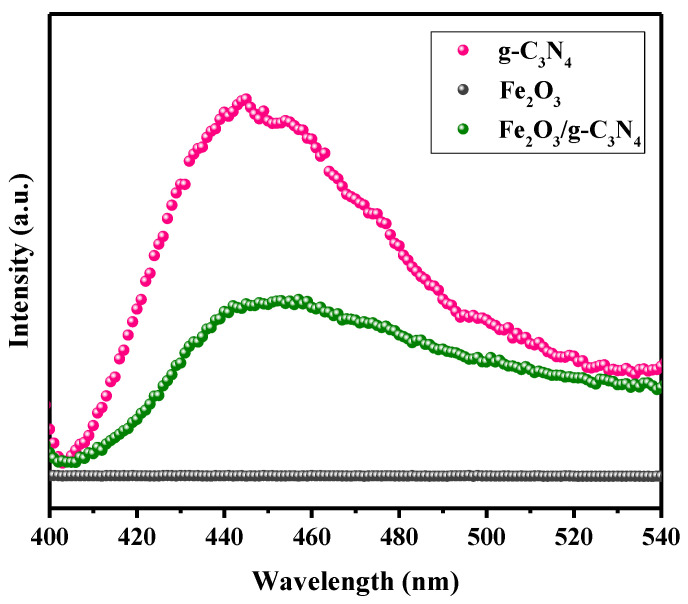
PL spectra of g–C_3_N_4_, Fe_2_O_3_ and Fe_2_O_3_/g–C_3_N_4_ catalysts.

The separation efficiency and transfer behavior of photogenerated carriers can be investigated by photoluminescence (PL) spectra. In general, a reduced PL spectra signal is attributed to a decreased combination rate of photogenerated electron–hole carriers, bringing about an enhanced photocatalytic performance [35]. As shown in Figure 5, it is observed that the fluorescence signals of the Fe_2_O_3_/g–C_3_N_4_ composite are notably weaker than that of pure g–C_3_N_4_, indicating that Fe_2_O_3_ could capture the photogenerated electrons from g–C_3_N_4_, leading to effective improvements in photogenerated electron–hole pair separation. No prominent emission peak for Fe_2_O_3_ was observed in our work, which is consistent with other reports [25,35,36].

**Figure 6:**
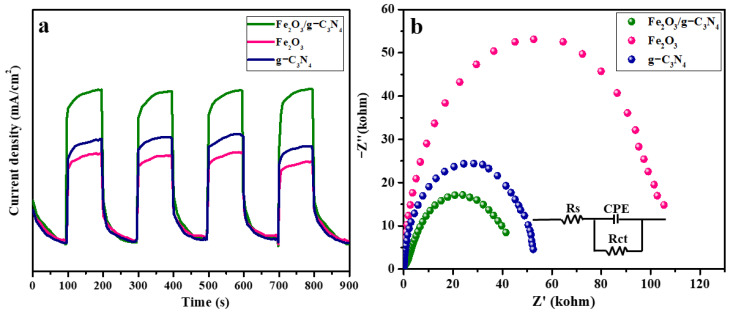
The i–t curves (**a**) and EIS spectra (**b**) of as-prepared samples.

The migration and separation properties of the photogenerated carriers of g–C_3_N_4_, Fe_2_O_3_ and Fe_2_O_3_/g–C_3_N_4_ samples were further evaluated by transient photocurrent response (i–t) curves and impedance electrochemical spectroscopy (EIS). An intercept at a high-frequency area corresponds to the ohmic resistance (Rs) of the electrode and electrolyte, and a semicircle is ascribed to charge transfer resistance (Rct), while a straight line (CPE) is related to the ion diffusion at the electrode. As shown in Figure 6, Fe_2_O_3_/g–C_3_N_4_ exhibits a stronger photocurrent response and a smaller impedance arc radius relative to that of g–C_3_N_4_ and Fe_2_O_3_, indicating that the resistance to charge migration is greatly reduced, while the charge separation is effectively improved, by the construction of a heterojunction between Fe_2_O_3_ and g–C_3_N_4_, yielding excellent photocatalytic activity.

### 2.6. Photocatalytic Performance

**Figure 7:**
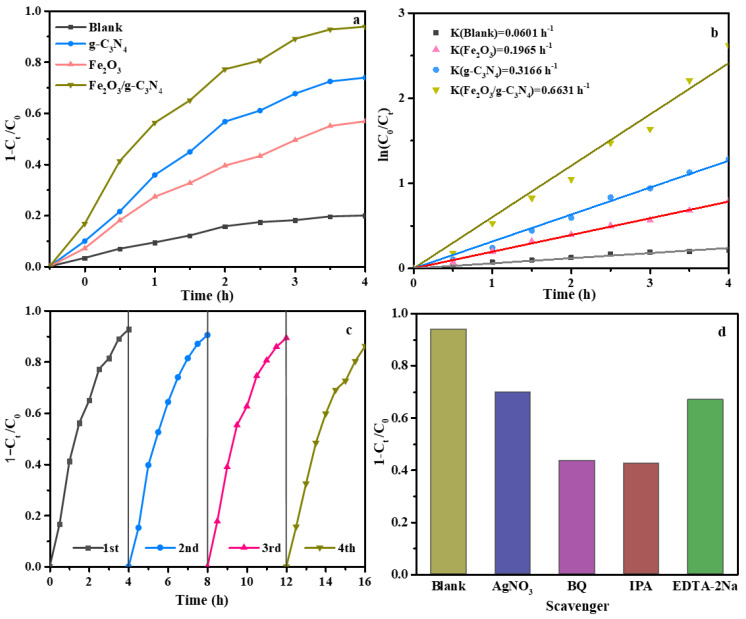
Degradation of NOR with g–C_3_N_4_, Fe_2_O_3_ and Fe_2_O_3_/g–C_3_N_4_ (**a**), the corresponding pseudo–first-order kinetics of NOR degradation (**b**), recycling stability of the Fe_2_O_3_/g–C_3_N_4_ sample (**c**) and the trapping test of the Fe_2_O_3_/g–C_3_N_4_ composite (**d**).

The photocatalytic activities of the synthesized catalysts were assessed through the degradation of NOR under visible light irradiation. The results of the blank experiments in Figure 7a show that low photocatalytic activity was observed in the absence of light, suggesting the NOR degradation is induced by photocatalysis. The degradation efficiency of NOR with the g–C_3_N_4_, Fe_2_O_3_ and Fe_2_O_3_/g–C_3_N_4_ catalysts was 74.2%, 56.7% and 94.7%, respectively. These results demonstrate that the photocatalytic activity of the Fe_2_O_3_/g–C_3_N_4_ composite was enhanced compared to g–C_3_N_4_ or Fe_2_O_3_. As displayed in the SEM and TEM, the perfect heterojunction interface was formed between g–C_3_N_4_ and Fe_2_O_3_, resulting in the high transportation and separation of charges and further enhancement of photocatalytic performance.

The reaction kinetics of NOR photo-degradation processes in the presence of g–C_3_N_4_, Fe_2_O_3_ and Fe_2_O_3_/g–C_3_N_4_ were fitted by the equation Ln(*C_0_*/*C_t_*) = *k*t (where *C_0_* and *C_t_* are the NOR concentrations at *0* and *t* hours, *k* is the kinetic constant and *t* is the reaction time). As shown in Figure 7b, the first-order kinetic constant *k* value for the Fe_2_O_3_/g–C_3_N_4_ composite is 0.6631 h^−1^, which is 3.37 and 2.09 times higher than that of the pure Fe_2_O_3_ (0.1965 h^−1^) and g–C_3_N_4_ (0.3166 h^−1^) samples, respectively. A comparison of the efficiency and kinetic results obtained from the as-prepared catalysts with other results in the literature are listed in Table 1. It was found that the prepared Fe_2_O_3_/g–C_3_N_4_ composite showed better NOR degradation when compared with BiOCOOH/O–gC_3_N_4_/CTS [37], La–BiFeO_3_ [38], BiVO_4_@LDHs [39] and CeVO_4_/BiVO_4_ [40].

**Figure 8:**
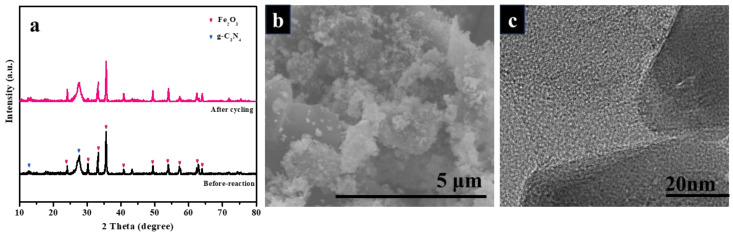
XRD patterns of Fe_2_O_3_/g–C_3_N_4_ after cycling (**a**). SEM (**b**) and TEM (**c**) images of Fe_2_O_3_/g–C_3_N_4_ post-reaction.

To investigate the recyclability and stability of the Fe_2_O_3_/g–C_3_N_4_ composite, its cycling performance was explored, and the results are shown in Figure 7c. After four cycles, NOR photo-degradation was still 86.7%, indicating that no critical photo-corrosion or self-degradation existed during the photocatalytic process. As shown in Figure 8a, the diffraction peaks of the Fe_2_O_3_/g–C_3_N_4_ composite did not change significantly after cycling. As shown in Figure 8b,c, Fe_2_O_3_ particles were still fixed onto the layered structure of g–C_3_N_4_, further confirming the stability of the prepared catalyst.

### 2.7. Photocatalytic Degradation Mechanism

A trapping test of the Fe_2_O_3_/g–C_3_N_4_ composite was carried out to determine the active species in the NOR photo-degradation process. As shown in Figure 7d, the photo-degradation of NOR is decreased from 94.1% to 43.8%, 42.9%, 66.8% and 70.1% with the addition of BQ, IPA, EDTA–2Na and AgNO_3_, respectively. Therefore, all four active species of •O_2_^−^, •OH, h^+^ and e^−^ affect NOR degradation, while the main active radicals are •OH and •O_2_^−^.

**Figure 9:**
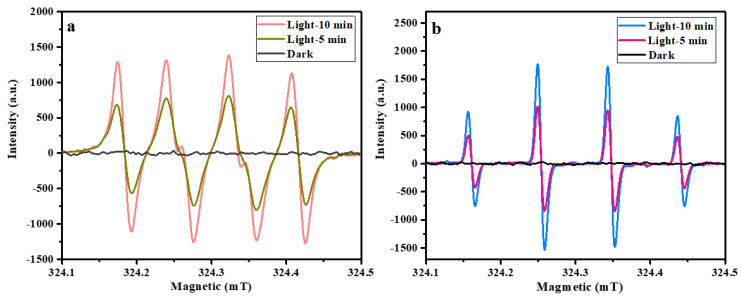
ESR signals of •O_2_^−^ (**a**) and •OH (**b**) in darkness and with visible light irradiation.

To further confirm the activity of •O_2_^−^ and •OH radicals during photo-degradation of the NOR system, the ESR technique was performed on the Fe_2_O_3_/g–C_3_N_4_ heterojunction using 5, 5-dimethyl-1-piropride-N-oxide (DMPO) as a trapper. As shown in Figure 9a,b, no characteristic signals of DMPO–•OH or DMPO–•O_2_^−^ were detected under dark conditions. However, the relative intensity ratios of 1:1:1:1 (Figure 9a) and 1:2:2:1 (Figure 9b) were observed under visible light irradiation, which correspond to the characteristic peaks of DMPO–•OH and DMPO–•O_2_^−^, respectively. In addition, the characteristic peaks of DMPO–•OH and DMPO–•O_2_^−^ increased with an increase in light irradiation time from 5 min to 10 min, indicating the generation of •O_2_^−^ and •OH radicals during photo-degradation of the NOR system. Of note, the signal strength of DMPO–•OH is similar to DMPO–•O_2_^−^, suggesting that both •OH and •O_2_^−^ are the major reactive species for NOR photogeneration, which is consistent with the conclusions of the trapping experiments.

**Figure 10:**
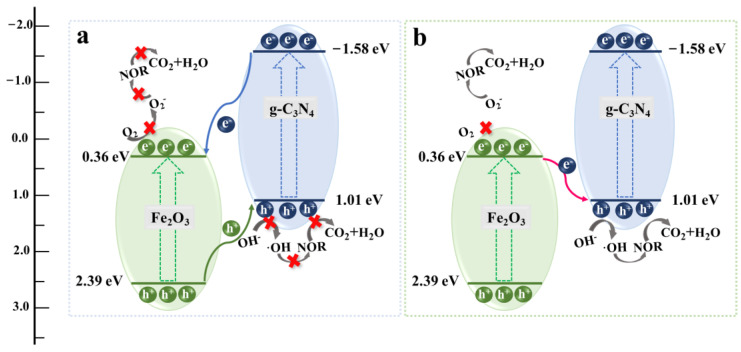
Possible mechanism of NOR photo-degradation by Fe_2_O_3_/g–C_3_N_4_ composites: (**a**) type II and (**b**) S-scheme.

Based on the above results and discussion, two possible photocatalytic degradation mechanisms for NOR with Fe_2_O_3_/g–C_3_N_4_ composites are proposed, including type II and S-scheme heterojunctions. Under visible light illumination, photogenerated electrons (e^−^) on the VB of Fe_2_O_3_ and g–C_3_N_4_ are transferred to the CB, and holes (h^+^) are left in the VB. In addition, the VB potential of Fe_2_O_3_ is higher than that of g–C_3_N_4_ and the CB potential of Fe_2_O_3_ is higher than that of g–C_3_N_4_. According to the type II electron transfer mode in Figure 10a, electrons transfer from the CB of g–C_3_N_4_ to the CB of Fe_2_O_3_, while holes transfer from the VB of Fe_2_O_3_ to the VB of g–C_3_N_4_. The CB energy of Fe_2_O_3_ (0.36 eV) is higher than O_2_/•O_2_^−^ (−0.33 eV) and the VB of g–C_3_N_4_ (1.01 eV) is lower than OH^−^/•OH (1.90 eV). Therefore, the electrons in the CB of Fe_2_O_3_ and the holes in the VB of g–C_3_N_4_ cannot react with dissolved/adsorbed O_2_ and H_2_O/OH^−^ molecules to form •O_2_^−^ and •OH, respectively. It is obvious that the traditional charge transfer mode is not consistent with the free radical trapping and ESR results, indicating that the type II electron transfer mode is not applicable in as-prepared Fe_2_O_3_/g–C_3_N_4_ composites in this study. The S-scheme electron transfer mode is proposed and shown in Figure 10b. Under irradiation, photogenerated electrons jump from the CB of Fe_2_O_3_ to the VB of g–C_3_N_4_ and rapidly combine with the holes in the VB of g–C_3_N_4_, leading to the accumulation of electrons with a higher reducing ability in the CB of g–C_3_N_4_ and to the retention of holes with a higher oxidation ability in the VB of Fe_2_O_3_. Importantly, adsorbed/dissolved O_2_ could be activated into •O_2_^−^ by electrons with higher reducing abilities, and OH^−^/H_2_O could be oxidized to •OH by holes with higher oxidation abilities. Eventually, the reaction species of •O_2_^−^, •OH, e^−^(g–C_3_N_4_) and h^+^ (Fe_2_O_3_) could degrade NOR into CO_2_ and H_2_O in a process that can be described as follows:

g–C_3_N_4_ + *hv* → e^−^ + h^+^ (4)

Fe_2_O_3_ + *hv* → e^−^ + h^+^ (5)

e^−^(Fe_2_O_3_) + h^+^(g–C_3_N_4_) → Recombined (6)

e^−^(g–C_3_N_4_) + O_2_ → •O_2_^−^ (7)

h^+^(Fe_2_O_3_) + OH^−^/H_2_O → •OH (8)

•OH/•O_2_^−^/h^+^(Fe_2_O_3_)/e^−^(g–C_3_N_4_) + NOR → CO_2_ + H_2_O (9)

Possible NOR photo-degradation with bare Fe_2_O_3_ or g–C_3_N_4_ is proposed as follows: Firstly, photogenerated electrons (e^−^) on the VB of Fe_2_O_3_ and g–C_3_N_4_ are transferred to CB and holes (h^+^) are left in the VB under visible light illumination. Then some of photogenerated electrons are readily recombined with holes. For bare g–C_3_N_4_, unrecombined electrons react with dissolved/adsorbed O_2_ to form •O_2_^−^; however, unrecombined holes cannot react with H_2_O/OH^−^ molecules to form •OH. For bare Fe_2_O_3_, unrecombined holes could react with H_2_O/OH^−^ molecules to form •OH, and unrecombined electrons cannot react with dissolved/adsorbed O_2_ to form •O_2_^−^. Based on the above comparison, it was discovered that the reaction species and behavior involved in the photo-degradation of NOR by S-scheme Fe_2_O_3_/g–C_3_N_4_ are more complicated than that of bare Fe_2_O_3_ or g–C_3_N_4_.

## 3. Experiment

### 3.1. Synthesis of Fe_2_O_3_/g–C_3_N_4_ Composites

The g–C_3_N_4_ was prepared by the thermal polymerization method [25]. According to this method, 0.03 mol of melamine was poured into a covered crucible and heated at 550 °C for 4 h with a heating rate of 10 °C∙min^−1^.

The Fe_2_O_3_ was prepared by a simple hydrothermal method [23]. First, 0.01 mol of FeCl_3_∙6H_2_O was added to an ethanol solution (50 mL anhydrous ethanol and 5 mL deionized water) and stirred continuously until the FeCl_3_∙6H_2_O was completely dissolved. Second, 0.01 mol of CH_3_COONa was added to the above mixed solution while vigorously stirring for 1 h. Third, the aforementioned dispersion was transferred into a 100 mL Teflon-lined stainless steel autoclave and subjected to heat treatment at 180 °C for 6 h. Finally, the obtained products were centrifuged, washed with deionized water and ethanol and dried at 100 °C for 12 h.

The Fe_2_O_3_/g–C_3_N_4_ composite was synthesized by an ultrasonic assistance method. First, 2.0 g of as-prepared g–C_3_N_4_ and 0.4 g of as-prepared α-Fe_2_O_3_ were mixed uniformly. Then the mixture and 5 mL of ethanol were added into a 10 mL glass tube, which was put into an ultrasonic bath (KQ-800E, 500 × 300 × 200, 40 KHz, Kunming, China) with a constant heating temperature function to maintain the temperature at 50 °C for 120 min. Finally, the powder was washed with ethanol and dried at 100 °C for 12 h.

### 3.2. Characterization

The crystal structures of as-synthesized samples were analyzed by X-ray diffraction (XRD, Bruker D8 Advance, Karlsruhe, Germany). The morphology and microstructure of the photocatalysts were studied by scanning electron microscope (SEM, SU8010, Tokyo, Japan) and transmission electron microscopy (TEM, Tecnai G2 F30 S-TWIN, Ann Arbor, MI, USA). The surface chemical compositions of samples were determined via energy dispersive spectroscopy combined with TEM (TEM–Mapping) and X-ray photoelectron spectroscopy (XPS, JPS-9010, Tokyo, Japan). The light absorption properties of the products were investigated by UV–vis diffuse reflectance spectra (DRS, UV-3600, Tokyo, Japan). The photoluminescence (PL) spectra were obtained at room temperature on a FLS1000 spectrophotometer (Edinburgh Instruments Ltd., Livingston, UK). The i–t curves and electrochemical impedance spectra (EIS) were evaluated using a CHI 660 E electrochemical analyzer (Chenhua, Shanghai, China) in a typical three-electrode configuration with a FTO glass substrates (2 cm × 2 cm) modified with as-prepared samples as the working electrode, a Pt foil as the counter electrode and an Ag/AgCl electrode as the reference electrode. In addition, 0.2 M Na_2_SO4 was used as the electrolyte solution, and a 250 W Sodium lamp (Chenhua, Shanghai, China) was utilized as the light source for the photoelectrochemical measurements. Electron spin resonance (ESR, JEOL JES-FA200, Akishima, Japan) was employed to detect active radicals of •O_2_^−^ and •OH with 5,5-dimethyl-1-pyrroline-*N*-oxide (DMPO) as a capture agent.

### 3.3. Photocatalytic Experiment

Firstly, 50 mg of the prepared catalyst (Fe_2_O_3_, g–C_3_N_4_, Fe_2_O_3_/g–C_3_N_4_) was dispersed in 200 mL of a 10 mg∙L^−1^ NOR aqueous solution and stirred in a dark environment for 60 min to ensure an adsorption–desorption equilibrium. Then, this was irradiated by a 250 W sodium lamp with recirculating cooling water. During the degradation experiment, about 5 mL of the suspension was dropped out at regular intervals and centrifuged to remove the solid catalyst by high-speed centrifugation. Finally, the concentration of the residual NOR was monitored by UV–vis absorption spectroscopy at a wavelength of 273 nm.

In order to determine the dominant reactive species, benzoquinone (BQ), isopropanol (IPA), ethylenediaminetetraacetic acid disodium salt (EDTA–2Na) and silver nitrate (AgNO_3_) were added to the degradation experiment to remove active species of •O_2_^−^, •OH, h^+^ and e^−^, respectively.

## 4. Conclusions

In this work, S-scheme Fe_2_O_3_/g–C_3_N_4_ heterojunctions were successfully prepared by the microwave assistance method, which exhibited enhanced photocatalytic activity in the degradation of NOR under visible light irradiation. Photocatalytic degradation of NOR with as-prepared Fe_2_O_3_/g–C_3_N_4_ was found to be about 1.67 and 1.28 times higher than that of pure Fe_2_O_3_ and g–C_3_N_4_, respectively. The kinetic constant of NOR removal with the Fe_2_O_3_/g–C_3_N_4_ composite was about 0.6631 h^−1^, and NOR photo–degradation was still 86.7% after four cycles. These improvements were primarily attributed to the composite’s enhanced light absorption and effective separation of photogenerated charges. Furthermore, the trapping experiments and ESR results demonstrated that •O_2_^−^ and •OH were the primary radicals used for NOR photo-degradation, and a photo-degradation pathway of NOR with Fe_2_O_3_/g–C_3_N_4_ was proposed. Hence, S-scheme heterojunction photocatalysts have outstanding application potential in the treatment of water pollution.

## Figures and Tables

**Table 1:** Study results in comparison with other photocatalysts in the literature.

Photocatalyst	Degradation Efficiency (%)	Degradation Rate Constant	Reference
Fe_2_O_3_/g–C_3_N_4_	94.7%	0.6631 h^−1^	This work
BiOCOOH/O–gC_3_N_4_/CTS	82.1%	0.02543 min^−1^	[37]
La–BiFeO_3_	84.94%	0.01638 min^−1^	[38]
BiVO_4_@LDHs	90.3%	0.02078 min^−1^	[39]
CeVO_4_/BiVO_4_	84.8%	0.3618 h^−1^	[40]

## Data Availability

Data are contained within the article.
